# Nucleus pulposus clamping procedures based on optimized material point method for surgical simulation systems

**DOI:** 10.1186/s42492-025-00188-9

**Published:** 2025-04-01

**Authors:** Jianlong Ni, Jingrong Li, Zhiyuan Xie, Qinghui Wang, Chunhai Li, Haoyu Wu, Yang Zhang

**Affiliations:** 1 School of Mechanical and Automotive Engineering, South China University of Technology, Guangzhou 510640, Guangdong China; 2 Guangdong Artificial Intelligence and Digital Economy Laboratory (Guangzhou), Guangzhou 510335, Guangdong China; 3 Department of Orthopaedics, Sun Yat-Sen Memorial Hospital, Sun Yat-Sen University, Guangzhou 510120, Guangdong China; 4 Department of Psychology, Soochow University, Suzhou 215000, Jiangsu China

**Keywords:** Material point method, Transforaminal endoscopic lumbar discectomy, Force feedback, Surgical training

## Abstract

Clamping and removal of the nucleus pulposus (NP) are critical operations during transforaminal endoscopic lumbar discectomy. To meet the challenge of simulating the NP in real-time for better training output, an improved material point method is proposed to represent the physical properties of the NP and compute its deformation in real time. Corresponding volume rendering of the NP and its hosting bones are also presented. The virtual operation procedures are then implemented into a training prototype and subsequently tested through simulation experiments and subjective evaluation. The results have demonstrated the feasibility of the approach.

## Introduction

Transforaminal endoscopic lumbar discectomy (TELD) surgery has been an effective minimally invasive alternative to open surgery due to its numerous benefits, resulting in its increasing application [1]. However, the success of TELD heavily relies on the surgeon’s surgical skills and expertise, which leads to the disadvantage of long learning curves for the procedure [2]. Based on surgical data statistics, at least 20 cases of surgery are required to achieve proficiency in performing such procedures [3]. Additionally, the key step in TELD is the removal of the nucleus pulposus (NP), which may sometimes cause insufficient removal, leading to recurrence and side effects [2]. Therefore, the clamping and removal of the NP necessitates extra training and practice to ensure effective results.

Virtual simulation training has been proven to effectively shorten the learning curve for physicians, demonstrating valuable support in their training simulations [4, 5]. Moreover, a virtual surgical simulation training system with integrated force feedback interaction technology provides a better solution for surgeons to practice. In recent years, force feedback technology has been increasingly applied in various types of surgeries, particularly in the simulation training of minimally invasive procedures [6–8]. Nevertheless, the research and work on integrating force feedback functionality in the TELD virtual simulation system based on virtual reality (VR) technology are relatively lacking [9, 10].

The primary challenge of virtual training with force feedback is how to precisely simulate NP, the core operative element in TELD. While rendering of the resulting force on NP after virtual surgical actions goes beyond merely visualization, and is closely intertwined with real-time physics simulation of the model. Commonly used methods of modeling can be broadly categorized into two: mesh-based and meshless [11]. The mesh-based methods commonly employed include the mass-spring model [12], finite element method [13], position based dynamics [14], and projective dynamics [15], etc. While dominating meshless methods are the material point method (MPM) [16], smoothed particle hydrodynamics [17], and element-free Galerkin methods [18]. Mesh-based methods can provide more accurate simulations for objects without large deformation, but when the model needs to be greatly deformed or even damaged, corresponding calculations with mesh-based models are troublesome. In such cases, the advantages of the meshless method become prominent due to its ability to support free topological particle relationships.

As a biologically active material, NP comprises both solid and liquid components and possesses a certain degree of internal mobility [19]. According to observations from actual surgical procedures, the extracted material after TELD surgery is predominantly in a solid state. Hence, based on the analysis of the above algorithms and considering such material characteristics, a meshless method, more specifically, the MPM is more favorable for its modeling. In the realm of the meshless method, Bao et al. [20] used the three-parameter viscoelastic model to simulate stress-strain, stress relaxation, and creep properties. Shi et al. [21] use the meshless method for cutting simulation. In their work, the gradient of physical quantities such as displacement and velocity of the least square fitting point are used, but the touch model is with single point contact, and the force feedback algorithm is relatively simple.

Although VR and haptic/force feedback technologies have been well applied in some medical surgical procedures, they are relatively lacking in TELD training surgeries. In this work, a novel approach for simulating the clamping procedures for TELD surgical simulation is proposed. Due to the characteristics of the NP and its deformation up to four times or more during the procedure of the clamping operation, an optimized MPM-based model is used to achieve real-time simulation of the deformation of NP. The Ray-casting algorithm was used to visualize bones, and the marching cubes algorithm was used to visualize the NP to match the MPM. Subsequently, corresponding force calculations and rendering were integrated. The optimized MPM method takes into account the viscoelastic and plastic characteristics of the NP during virtual clamping operations, making the real-time simulation process closer to the actual changes in the NP after being clamped and deformed. At the same time, this method is also applicable to the real-time simulation of other biological soft tissue requiring simultaneous visual and haptic rendering, and can serve as a reference for the simulated manipulation of other soft-tissue organs.

## Methods

### The analysis of collision simulation between NP particles and the clamp

**Fig. 1:**
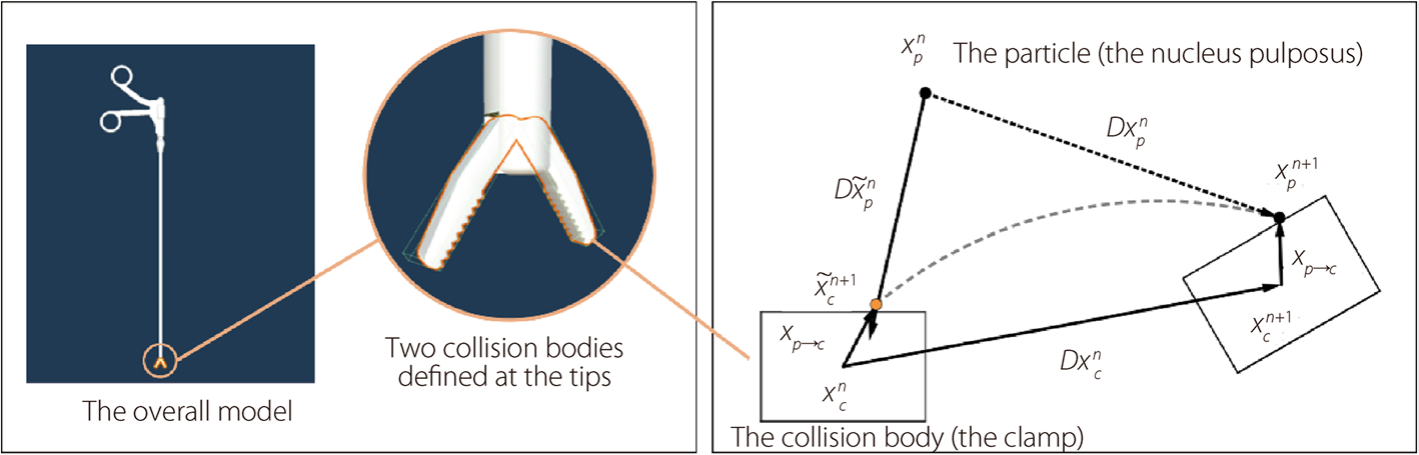
The modeling of the NP clamp and the updating of the NP particles’ position

In this paper, “the particles” refers to the fundamental units used in the simulation of the NP via the MPM algorithm. As shown in Fig. 1, it is sufficient to place two cubic collision boxes at the tip of the clamp to calculate its interaction with the NP. Then the calculation process is divided into simulation frames, denoted by *n*, *n* + 1, and so on, at fixed time intervals. ${D\widetilde{x}}_{p}^{n}$ represents the displacement of the particle. The particle and the clamp intersect at ${\widetilde{x}}_{p}^{n+1}$, and the relative position between them is denoted as ${x}_{p\to r}$. By adding the position of the clamp in the (*n* + 1)-th frame to their relative position, the position of the particle in the (*n* + 1)-th frame can also be determined and denoted as ${x}_{p}^{n+1}$.

### The constitutive modeling of the NP tissue

Due to the significant positional changes during the NP clamping process, the updated Lagrangian method is applied. The deformation gradient ${F}_{p}$ is used to measure local deformations of the object based on the current configuration. Hu et al. [22] suggested using the affine velocity matrix ${C}_{p}^{n}$ from the Affine Particle-In-Cell method [23] as an approximation to replace the tensor of the velocity. This approach can effectively accelerate the computation, hence it is adopted. Accordingly, the deformation gradient can be approximated as follows:

1 $${F}_{p}^{n+1}=(I+\Delta t{C}_{p}^{n}){F}_{p}^{n}$$

where the deformation gradients ${F}_{p}^{n}$ correspond to the deformation states of the particles at the *n*-th frame. $\Delta t$ is the time step length set for the simulation.

In the MPM, the momentum of the grid is interpolated from the physical quantities of the particles within the interpolation range (referred to as neighborhood particles). The grid momentum consists of two parts: the interpolation of the momentum of neighboring particles, denoted as ${(mv)}_{i}^{n+1}\left(particle\right)$, and the change in momentum due to local stress, denoted as ${(mv)}_{i}^{n+1}\left(stress\right)$ [16]:

2 $${(mv)}_{i}^{n+1}\left(particle\right)={m}_{p}{v}_{p}^{n}+{m}_{p}{C}_{p}^{n}({x}_{i}-{x}_{p}^{n})$$

3 $${(mv)}_{i}^{n+1}\left(stress\right)=\frac{4\Delta t}{\Delta {t}^{2}}{V}_{p}^{0}P\left({F}_{p}^{n+1}\right){\left({F}_{p}^{n+1}\right)}^{T}({x}_{i}-{x}_{p}^{n})$$

In Eq. 2, ${m}_{p}{v}_{p}^{n}$ represents the momentum of the particle. It describes the interpolation of the momentum of neighboring particles in the grid, without considering the effects of local stress on the object. Equation 3 describes the change in momentum due to the stress experienced by the particles. Here, ${V}_{p}^{0}$ represents the grid volume, and since the grid does not undergo distortion from the Eulerian perspective, ${V}_{p}^{n}={V}_{p}^{0}$. Therefore, the initial grid volume can be directly used. $P\left({F}_{p}^{n+1}\right)$ is the first Piola-Kirchhoff (PK) stress generated under this deformation gradient. This stress tensor represents the stress per unit reference configuration.

For the Neo-Hookean model, the relationship between energy density function $\psi \left(F\right)$ and the deformation gradient *F* is expressed as [22]:

4 $$\psi \left(F\right)=\frac{\mu }{2}\left(tr\left({F}^{T}F\right)-d\right)-\mu \text{log}\left(J\right)+\frac{\lambda }{2}{log}^{2}(J)$$

where $tr( )$ represents the trace of a matrix, $\mu$ and $\lambda$ are the Lamé coefficients, which are parameters reflecting the material's ability to deform, *d* is the dimension of the space in which the object resides, and* J* is the determinant of the deformation gradient *F*, representing the volume scaling factor between the current and reference configurations. The term $\frac{\mu }{2}\left(tr\left({F}^{T}F\right)-d\right)$ reflects the material’s resistance to shear deformation, while the term $-\mu \text{log}\left(J\right)+\frac{\lambda }{2}{log}^{2}(J)$ reflects the material’s incompressibility. The energy density increases sharply to resist compression when the material volume decreases. By taking the partial derivative of the energy density function with respect to the deformation gradient *F*:

5 $$P\left(F\right)=\mu \left(F-{F}^{-T}\right)+\lambda \text{log}(J){F}^{-T}$$

In plastic constitutive models, the strain in the material should be divided into elastic strain and plastic strain [25].

6 $$\varepsilon ={\varepsilon }_{E}+{\varepsilon }_{P}$$

where $\varepsilon$ represents the total strain, ${\varepsilon }_{E}$ is the elastic strain, and ${\varepsilon }_{P}$ the plastic one. From the perspective of the deformation gradient, it can be derived that:

7 $$F={F}_{E}{F}_{P}$$

where *F* is the deformation gradient, ${F}_{E}$ is the elastic deformation gradient, and ${F}_{P}$ is the plastic deformation gradient. Regarding the method proposed by Stomakhin et al. [16] for simulating plastic materials, principal strains instead of principal stresses can be used when defining the plastic deformation criterion. Therefore, Eq. 4 can be revised as follows:

8 $$\psi \left(F\right)=\frac{\mu ({F}_{P})}{2}\left(tr\left({{F}_{E}}^{T}{F}_{E}\right)-d\right)-\mu ({F}_{P})\text{log}\left({J}_{E}\right)+\frac{\lambda ({F}_{P})}{2}{log}^{2}({J}_{E})$$

where ${J}_{E}$ is the determinant of the elastic deformation gradient ${F}_{E}$. The Lamé coefficients are no longer set as constants, but rather as functions related to the plastic deformation gradient. They are defined as follows:

9 $$\mu \left({F}_{P}\right)={\mu }_{0}{e}^{\xi (1-{J}_{P})}$$

10 $$\lambda \left({F}_{P}\right)={\lambda }_{0}{e}^{\xi (1-{J}_{P})}$$

In the above formula, ${J}_{P}$ is the determinant of the plastic deformation gradient ${F}_{P}$, ${\mu }_{0}$ and ${\lambda }_{0}$ are the initial Lamé coefficients, representing the material’s Lamé coefficients during elastic deformation, and $\xi$ is the plastic hardening coefficient.

Equations 8 to 10 describe the constitutive model for material plasticity. However, like many soft tissues in the human body, the real NP exhibits both plastic and viscoelastic properties. To simulate the NP, it is necessary to describe the viscoelastic characteristics of the material, which represents the relationship between the material’s stress, strain, and the rate of strain change. Intuitively, viscoelastic materials typically exhibit characteristics such as ‘creep’ and “stress relaxation.” Creep refers to the phenomenon where, if sudden constant stress is applied at time zero without prior stress, the strain in the material gradually increases over time under such stress conditions until it stabilizes. Stress relaxation, on the other hand, occurs when a sudden strain is applied to an unstrained material at time zero. If this strain is held constant, the stress in the material will gradually decrease over time until it stabilizes.

For the above needs, this paper proposes a novel optimization method for the MPM, used to simulate the NP. For the simulation of the viscoelastic and plastic characteristics of materials, this work adopts the following approach: first, define the constant compression limit ratio ${\theta }_{C}$ and the extension limit ratio ${\theta }_{S}$, hereby restricting the material’s elastic deformation to the range $[1-{\theta }_{C}, 1+{\theta }_{S}]$, It can be described as follows:

11 $${J}_{E}=\left\{\begin{array}{c}J+{k}_{c}(1-{\theta }_{C}-J), J\in (-\infty, 1-{\theta }_{C})\\ J , J\in [1-{\theta }_{C}, 1+{\theta }_{S}]\\ J-{k}_{s}(J-1-{\theta }_{S}), J\in (1+{\theta }_{S}, +\infty )\end{array}\right.$$

where ${k}_{c}$ and ${k}_{s}$ are constants that affect the material’s viscosity. Setting the material’s deformation in this way offers the following advantages: when the material exceeds its range of elastic deformation, the energy increment brought about by plastic deformation significantly decreases. Additionally, this model can simulate the stress hysteresis characteristic of viscoelastic materials. When the material deformation exceeds the range of $1+{\theta }_{S}$, let ${J}_{E}$ gradually decrease, thus allowing for stress relaxation while maintaining the strain in the model.

The deformation gradient can be represented as follows:

12 $${F}_{E}=\frac{{J}_{E}}{J}F$$

13 $${F}_{P}={F}_{E}^{-1}F$$

By combining Eqs. 11, 12, and 13 with Eq. 8, which represent the energy density function of viscoelastic materials under strain, the first PK stress at this location can be obtained by taking the partial derivative of the energy density function:

14 $$P\left(F\right)=\mu \left({F}_{P}\right)\left({F}_{E}-{{F}_{E}}^{-T}\right)+\lambda \left({F}_{P}\right)\text{log}({J}_{E}){{F}_{E}}^{-T}$$

### The optimized MPM modeling process

**Fig. 2:**
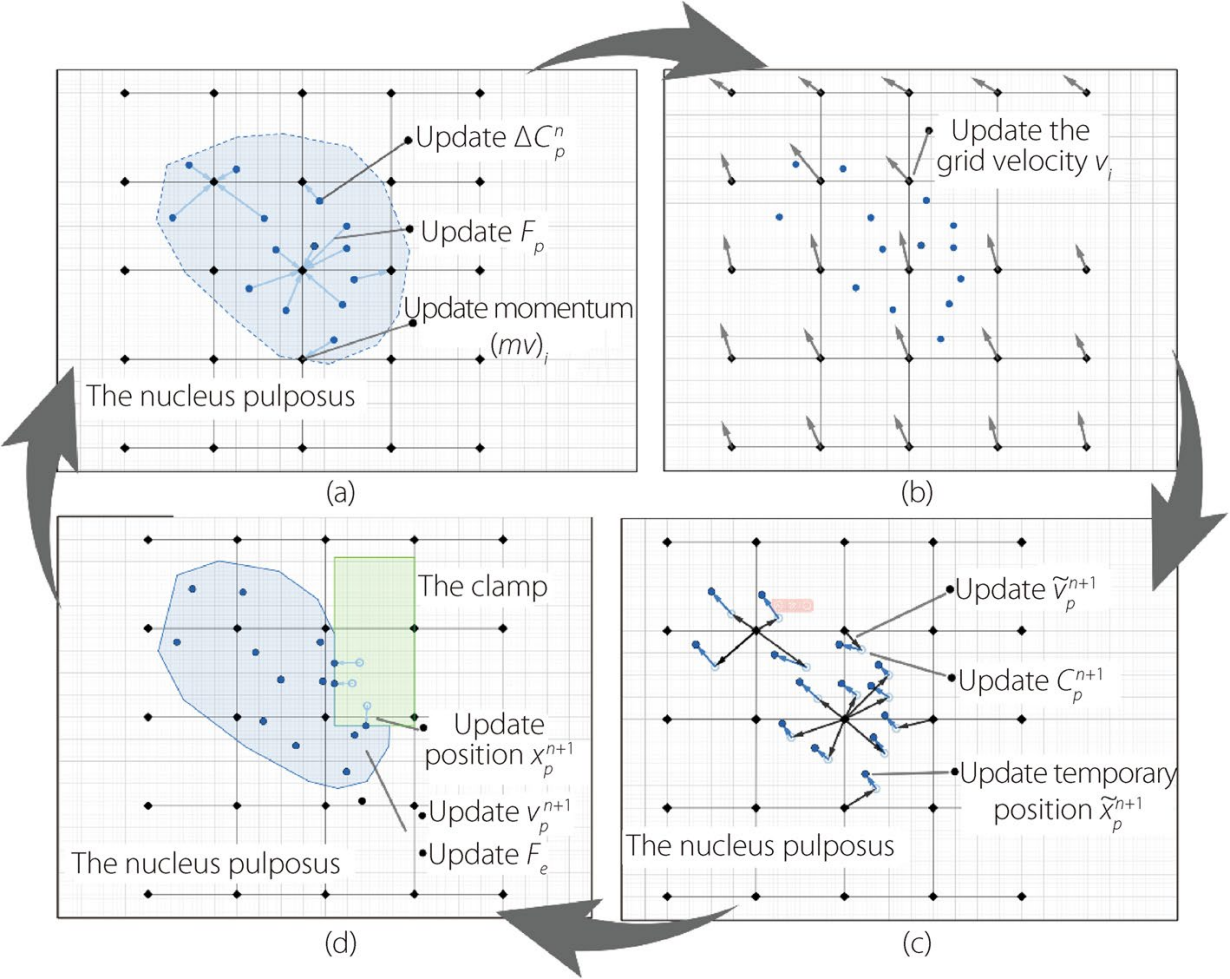
The optimized MPM procedure. **a** Particle to grid transfer; **b** Grid interaction;** c** Grid to particle transfer;** d** Update particle and force

Based on the analysis provided, the optimized MPM process suitable for simulating the NP clamping procedure can be divided into four steps, as shown in Fig. 2. The complete procedure is as follows:

#### Step 1. Particle to grid transfer

This step primarily involves interpolating physical information such as momentum and mass from the particles onto the grid nodes formed by partitioning the space where the NP may exist (referred to as the grid), as shown in Fig. 2a.

Update the increment of the affine velocity matrix ${\Delta C}_{p}^{n}$:

15 $$\Delta {C}_{p}^{n}=\frac{4}{\Delta {x}^{2}}\sum_{i}{\omega }_{ip}\Delta {v}_{i}^{n}{({x}_{i}-{x}_{p}^{n})}^{T}$$

where ${\omega }_{ip}$ is the interpolation function at the particle, which can use B-spline curves for interpolation, $\Delta {v}_{i}^{n}$ is the change in grid momentum within the MPM context, ${x}_{i}$ denotes the grid position, and ${x}_{p}^{n}$ is the position of the particle at the *n*-th frame.

Update the particle deformation gradient:

16 $${F}_{p}^{n+1}=(I+\Delta t({C}_{p}^{n}+{\Delta C}_{p}^{n})){F}_{p}^{n}$$

Update the grid momentum ${(mv)}_{i}^{n+1}$:

17 $${(mv)}_{i}^{n+1}=\sum_{p}{\omega }_{ip}\left\{{m}_{p}{v}_{p}^{n}+\left[{m}_{p}{C}_{p}^{n}-\frac{4\Delta t}{\Delta {x}^{2}}{V}_{p}^{0}P\left({F}_{p}^{n+1}\right){\left({F}_{p}^{n+1}\right)}^{T}\right]({x}_{i}-{x}_{p}^{n})\right\}$$

where $P(F)$ is the first PK stress tensor. $\Delta x$ is the grid spacing in MPM, and ${V}_{p}^{0}$ is the initial volume of the particle.

Update the grid mass ${m}_{i}^{n+1}$:

18 $${m}_{i}^{n+1}=\sum_{p}{m}_{p}{\omega }_{ip}$$

where ${m}_{p}$ represents the mass of the particle.

#### Step 2. Grid interaction

This mainly involves simulating the interaction between the object and other objects in the scene at the grid as shown in Fig. 2b.

According to Hu et al. [22], compute the temporary grid velocity ${\widetilde{v}}_{i}^{n+1}$:

19 $${\widetilde{v}}_{i}^{n+1}=\frac{{(mv)}_{i}^{n+1}}{{m}_{i}^{n+1}}$$

Update the grid velocity ${v}_{i}^{n+1}$ according to boundary conditions, that is, the velocity component towards the edge or obstacle of grid nodes and near edges or obstacles is set to zero:

20 $${v}_{i}^{n+1}=BC({\widetilde{v}}_{i}^{n+1})$$

where $BC({\widetilde{v}}_{i}^{n+1})$ represents the boundary condition treatment applied to the grid.

#### Step 3. Grid to particle transfer

This step primarily involves the interpolation of physical information from the grid back to the particles, as well as the interaction between the NP clamp and the NP at the particle level (as shown in Fig. 2c).

Update the temporary particle velocity ${\widetilde{v}}_{p}^{n+1}$:

21 $${\widetilde{v}}_{p}^{n+1}=\sum_{i}{\omega }_{ip}{v}_{i}^{n+1}$$

Update the affine matrix ${C}_{p}^{n+1}$:

22 $${C}_{p}^{n+1}=\frac{4}{\Delta {x}^{2}}\sum_{i}{\omega }_{ip}{v}_{i}^{n+1}{({x}_{i}-{x}_{p}^{n})}^{T}$$

Update the temporary particle position ${\widetilde{x}}_{p}^{n+1}$:

23 $${\widetilde{x}}_{p}^{n+1}= {x}_{p}^{n}+\Delta t{\widetilde{v}}_{p}^{n+1}$$

#### Step 4. Update particle and force

In this step, collision detection between the particles and the NP clamp is performed, and the positions and velocities of the particles are updated after the collision. Finally, the force calculations are completed (as shown in Fig. 2d).

Update the particle position ${x}_{p}^{n+1}$ and velocity ${v}_{p}^{n+1}$ after interacting with the NP clamp:

24 $${x}_{p}^{n+1}=Collide({\widetilde{x}}_{p}^{n+1})$$

25 $${v}_{p}^{n+1}= \frac{{x}_{p}^{n+1}-{x}_{p}^{n}}{\Delta t}$$

where $Collide(x)$ refers to the interaction between the NP clamp and the NP particles described in the previous sections.

Update the particle velocity increment ${\Delta v}_{p}^{n+1}$ and the grid velocity increment $\Delta {v}_{i}^{n+1}$ for *(n* + 1)-th frame:

26 $${\Delta v}_{p}^{n+1}={v}_{p}^{n+1}-{\widetilde{v}}_{p}^{n+1}$$

27 $${\Delta v}_{i}^{n+1}=\frac{1}{{m}_{i}}\sum_{p}{\omega }_{ip}{{m}_{p}\Delta v}_{p}^{n+1}$$

In Eq. 27, the change in the velocity of the NP particles is attributed to the change in momentum caused by the action of the clamp. Therefore, the impulse exerted by the clamp can be determined, which corresponds to the force output required by force feedback devices:

28 $${Force}_{output}=\sum_{p}-\frac{{m}_{p}({v}_{p}^{n+1}-{\widetilde{v}}_{p}^{n+1})}{\Delta t}$$

Based on the algorithm of the NP simulation described above, this work has constructed a TELD surgical simulation prototype system (TELD-SSPS) for virtual foraminotomy surgery training.

### Volume rendering of human bone and NP

**Fig. 3:**
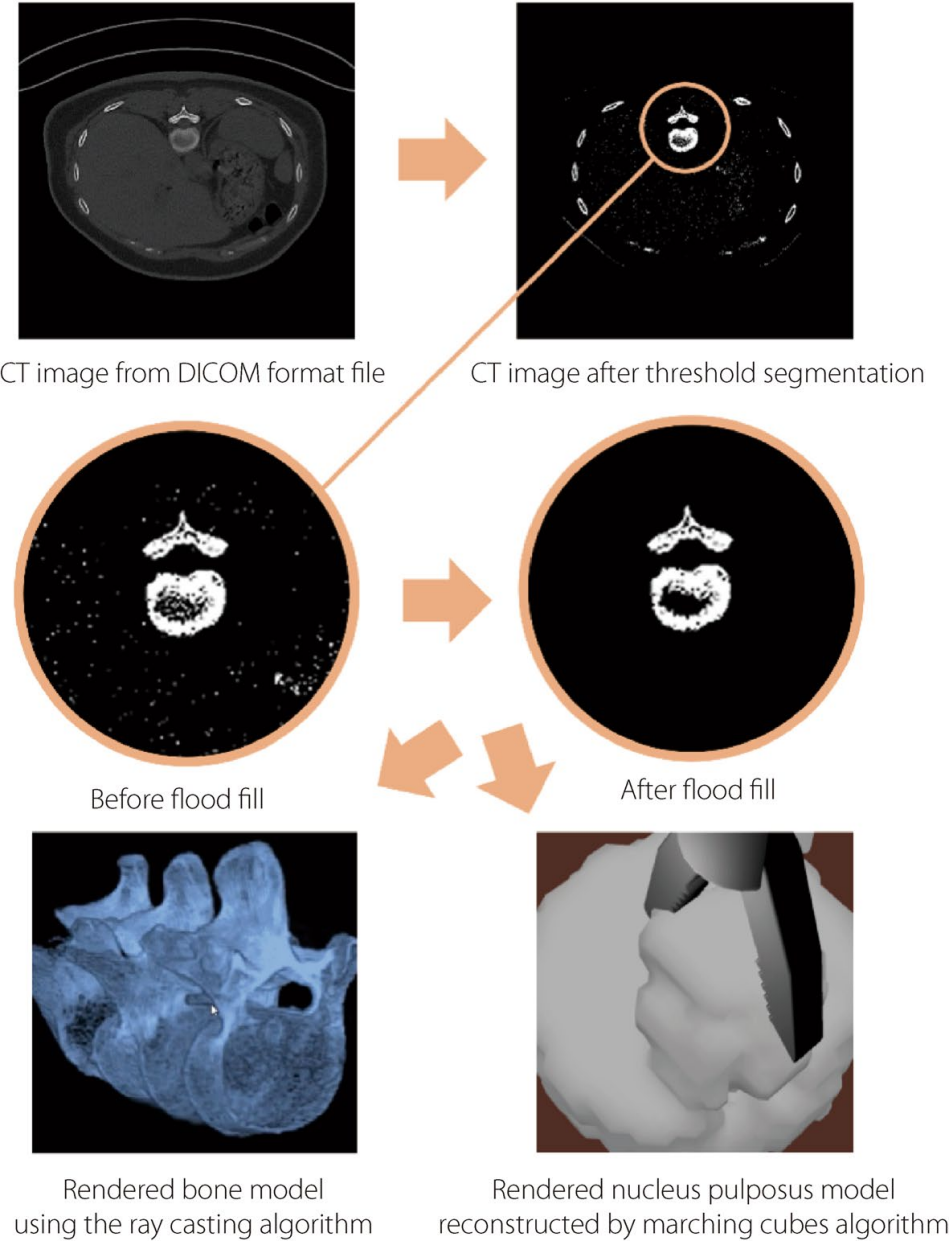
The processing procedure of volume rendering

To complement the particle-based simulation using MPM, a volume rendering technique is employed to achieve real-time display capabilities. To better cater to future medical applications, TELD-SSPS does not employ a fixed model but instead allows input of computed tomography images to generate corresponding human models. Since MPM employs particles for dynamic simulation, the direct volume rendering method is utilized to render models [24]. As shown in Fig. 3, threshold segmentation is employed to segment voxels of bone tissue. Then flood fill algorithms are applied to filter the image after segmentation. Finally, the ray-casting algorithm is applied for the reconstruction and rendering of human bones, and the marching cubes algorithm was used for voxelization, reconstruction, and rendering of the NP.

### System integration

**Fig. 4:**
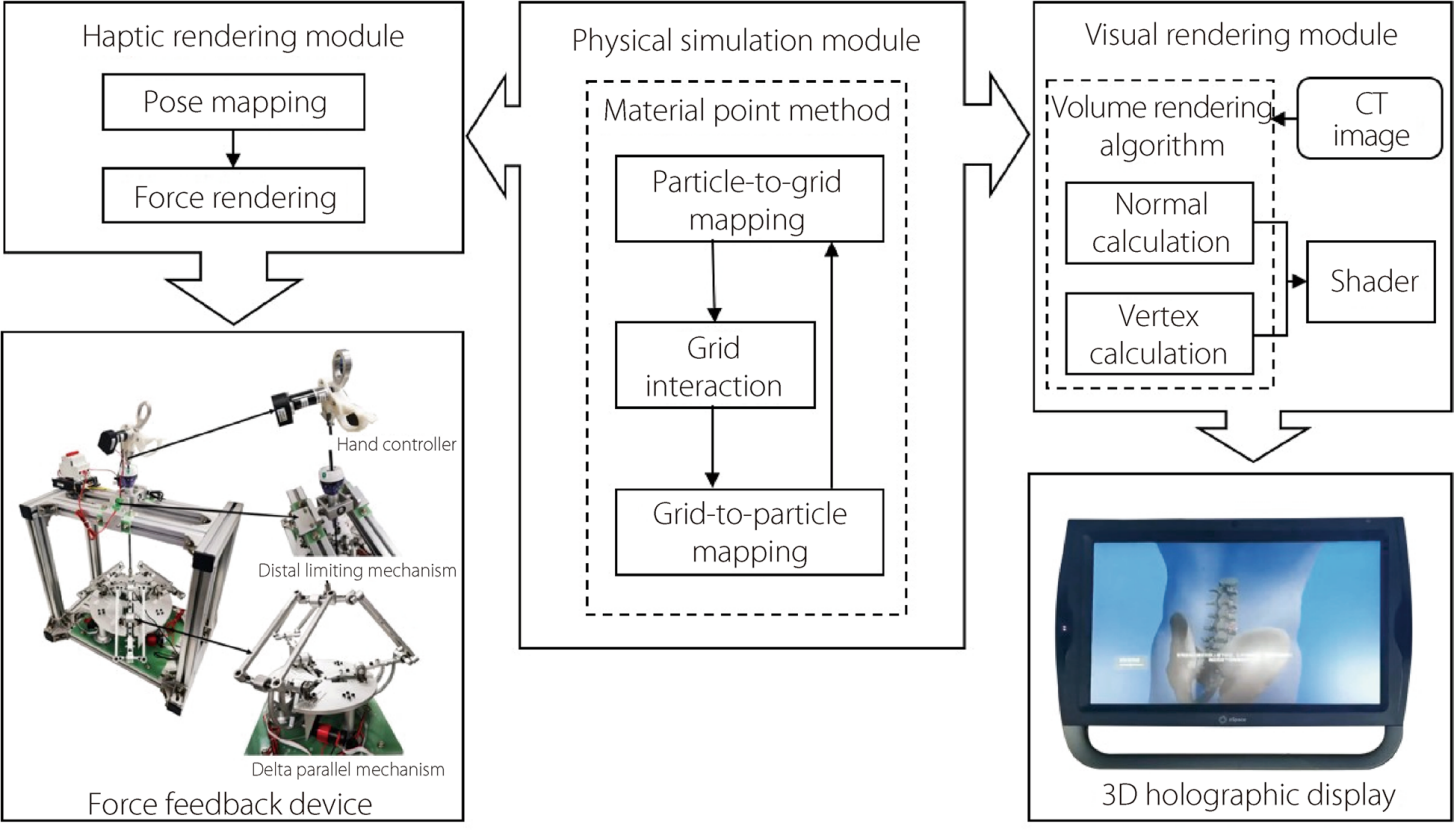
Three modules of the TELD-SSPSs

There are three modules in TELD-SSPS, shown in Fig. 4. The physical simulation module uses MPM to simulate the stress and strain of the NP. The force on the NP clamp calculated by MPM is transferred to the haptic rendering module. The algorithm takes into account GPU parallel computing to enhance its computational performance. For a better immersive experience, a holographic display (Zspace) is employed to provide a clearer three-dimensional visual effect. For haptic rendering, a six-DOF virtual surgery hand controller, developed by the authors’ research group, is used as the force feedback device.

### Validation of deformation characteristics of simulated NP tissue

A rectangular thin-layered NP simulation tissue, simulating the actual NP, is constructed by the methods proposed above. Experiments are designed to evaluate its three key characteristics for surgical operations.

### Design of the experiment

#### Plastic deformation characteristics

**Fig. 5:**
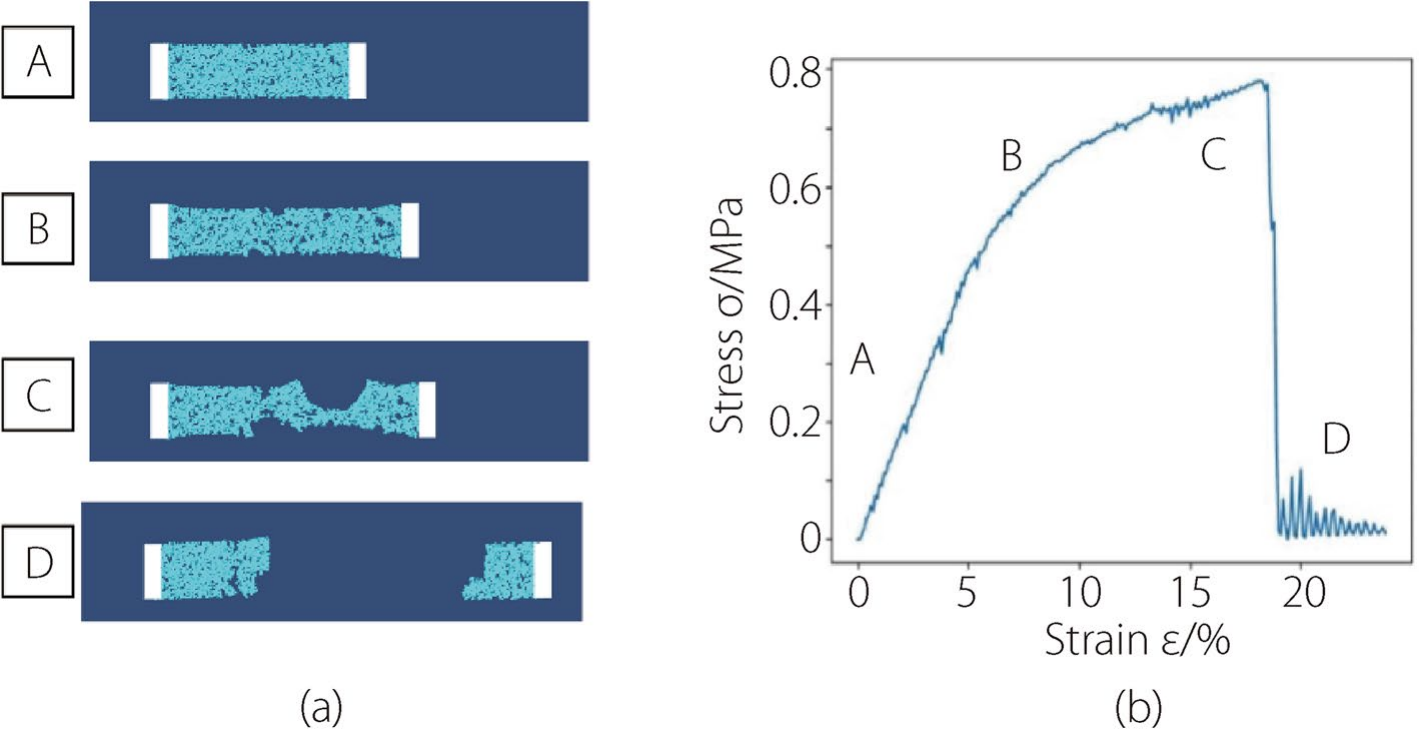
The simulation of plastic deformation characteristics. **a **The simulation of the tearing process; **b** The stress-strain curve during the tear simulation process

The simulated NP tissue is fixed at one end and pulled at a constant rate at the other end. This simulated tearing process, as shown in Fig. 5a, can be divided into four stages: the elastic deformation stage (stage A), where the stress and strain relationship is approximately linear; the plastic deformation stage (stage B), where the stress and strain relationship is no longer linear and partial cracks can be observed in the model; and stage C is tearing, where stress no longer increases but trembling, clear tears are visible on the model; while the last stage, stage D, the model is completely broken with the stress almost drops to zero. The oscillation of stress in stage D is due to the model suddenly breaking into two parts, causing the model to tremble. The occurrence of plastic deformation is indicated by a phase where the stress is stable and then abruptly changes, corresponding to a deformation exceeding 80% [25].

#### Creeping characteristics

Viscoelasticity refers to a condition that the stress applied to a material is not only related to the strain at the current moment but also to the rate of change of the strain [26]. To test whether such creeping characteristics can be well simulated, the left end of the NP simulation tissue is fixed and a constant stress is applied to the right end. During this process, the change of strain over time is observed.

#### Stress relaxation characteristics

For the simulation of stress relaxation characteristics of the NP tissue material, the left end of the NP simulation tissue is fixed and the right end is pulled to introduce a certain strain. This strain is then maintained, and the relationship between the internal stress and time from this moment is observed.

### Results

In the experiment for validating plastic deformation, the simulation result shown in Fig. 5c indicates that the modified MPM well simulates the tearing process of NP, replicating the process in which the model is torn beyond its elastic limit when subjected to external forces.

**Fig. 6:**
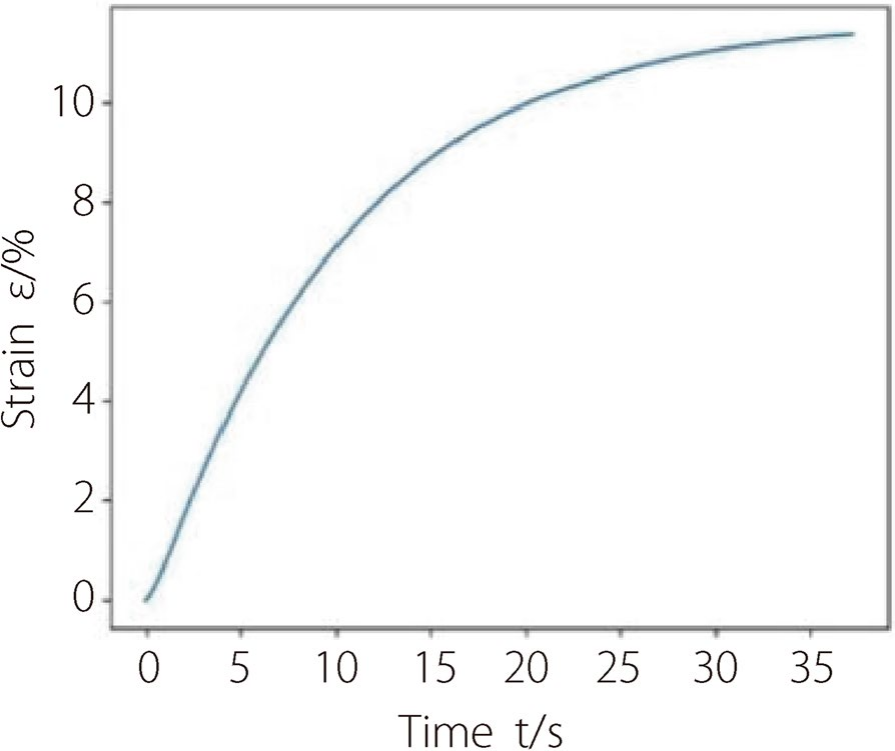
The time-strain relationship when testing creeping characteristic

While testing the creeping characteristic, the deformation exhibits a certain ‘lag’, showing time-dependent behavior. As shown in the time-strain curve (Fig. 6), the strain in the NP tissue increases over time with a rapid initial growth rate gradually approaching to steady state. This phenomenon is consistent with the actual creeping characteristics exhibited by soft tissues.

**Fig. 7:**
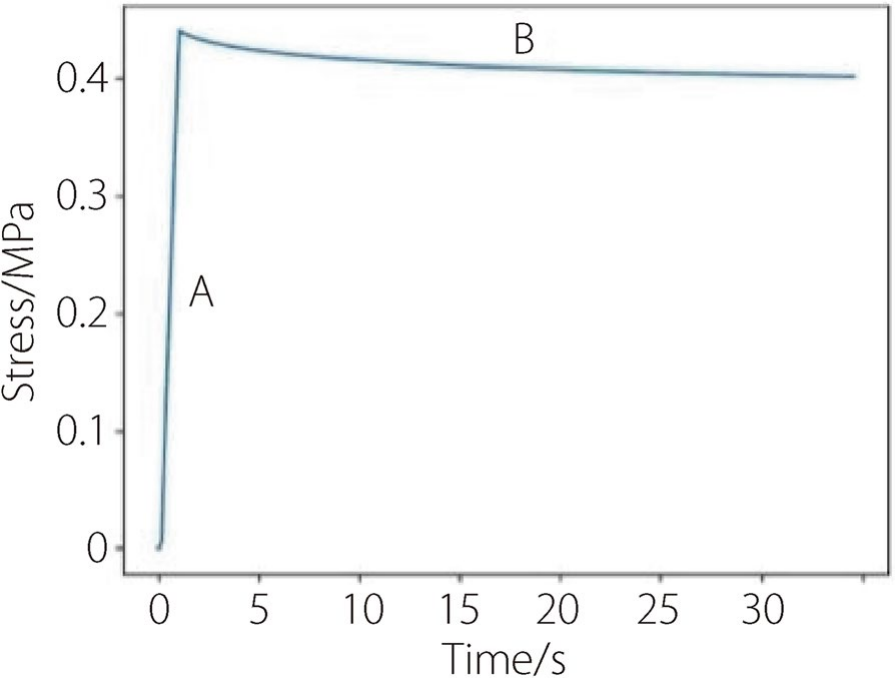
The time-stress relationship when testing stress relaxation

For the simulation of stress relaxation characteristics, the time-stress curve (Fig. 7) can be divided into two stages: A and B. In stage A, the soft tissue undergoes strain due to the application of external force. In stage B, the time stress is held constant and the stress decreases over time. Then the stress-decreasing rate slows down and eventually stabilizes. Throughout stage B, there is no change in strain, while the stress decreases. This stress relaxation behavior of the model validates the viscoelastic characteristic of the actual NP.

### Interactive performance evaluation of the TELD-SSPS

To better evaluate the prototype in assisting TELD training, an open recruitment in the collaborative hospital is conducted. Participants are required to have basic medical knowledge of TELD. Additionally, both novices and experienced surgeons are called for. After screening the applications, as shown in Table 1, 9 volunteers were recruited. Among them, 1–5 are fresh medical undergraduates, 6 and 7 are medical interns, and 8 and 9 are doctors more knowledgeable in actual TELD.

**Table 1:** Detailed information about the participants

Participant No.	Age group	Gender	Position	Familiarity with TELD
1	20–24	Female	Medical undergraduate	Unfamiliar
2		Male		
3				
4				
5				
6			Medical interns	Familiar
7	25–29			
8	45–49		Chief surgeons	
9	50–54			

**Fig. 8:**
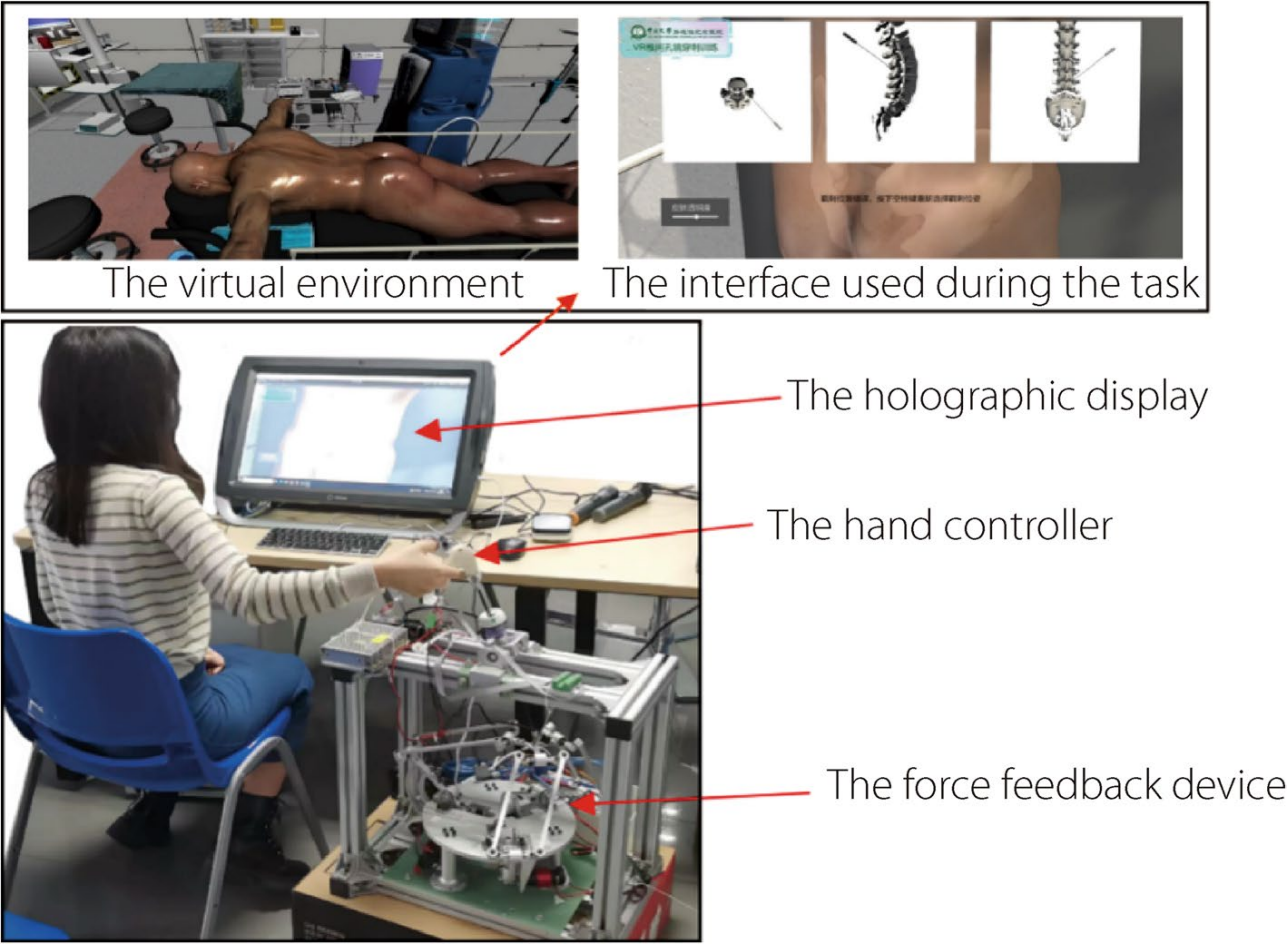
Experimental setup of TELD-SSPS

**Fig. 9:**
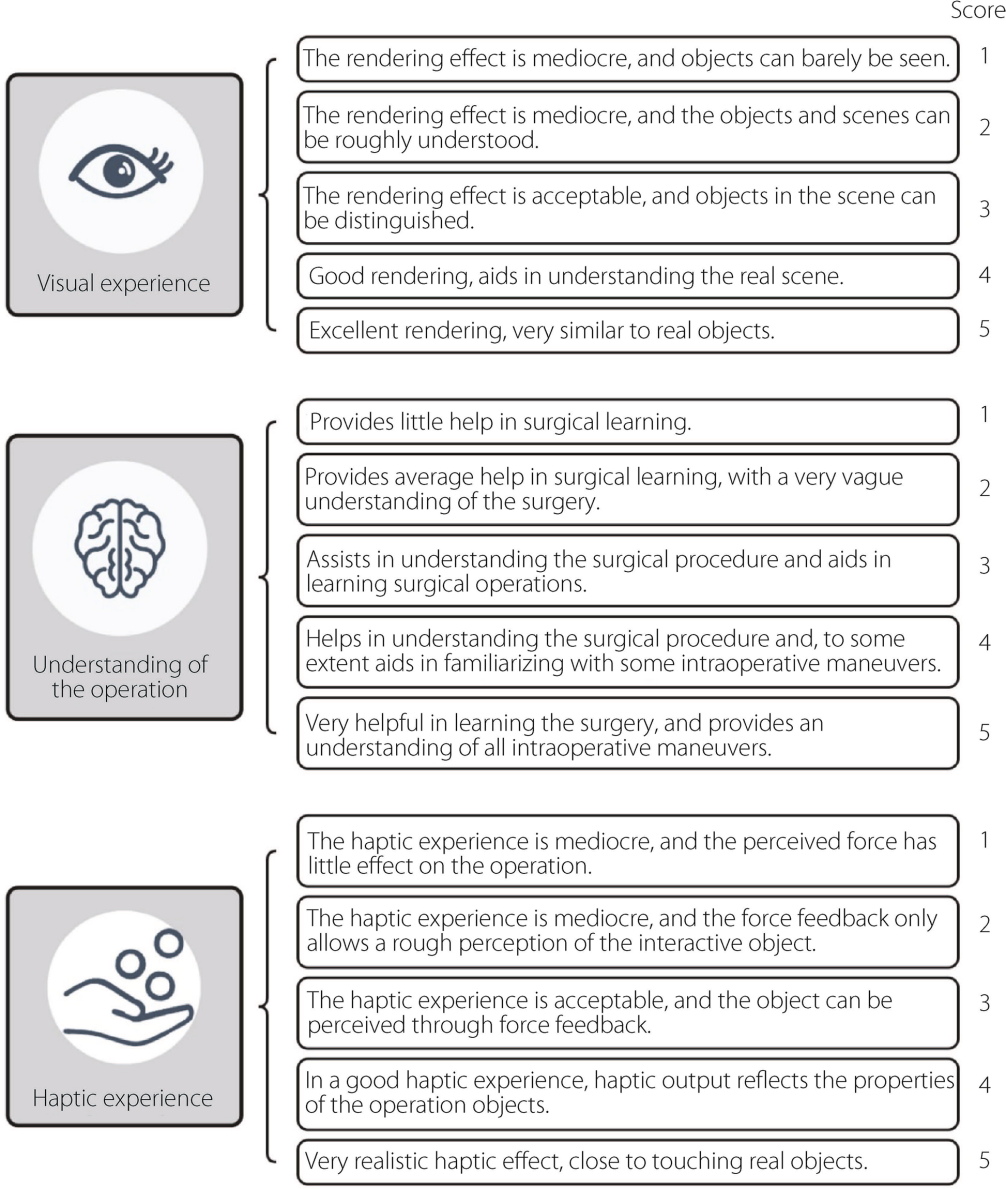
Scoring rules for TELD-SSPS

Before starting the formal experiments, each volunteer received individual training. This ensured that they understood the basic concepts of the procedure, mastered the right way of operating the force feedback device, and were clear about the goal of the task. Each volunteer then conducted at least five individual tests as shown in Fig. 8. After finishing the task, they are required to rate the prototype on visual experience, understanding of the surgery, and its haptic experience, according to the rules outlined in Fig. 9. Ratings below 3 require the participant to give a specific reason or explanation as feedback.

### Results

The subjective evaluation results are shown in Table 2.

**Table 2:** Subjective ratings of the prototype

Evaluation metric	Participant No.												
	1	2	3	4	5	Average of 1–5	6	7	Average of 6 and 7	8	9	Average of 8 and 9	Average
Visual experience	3	4	2	4	3	3.2	4	3	3.5	3	4	3.5	3.33
Understanding of the operation	5	4	3	4	4	4	4	3	3.5	3	3	3	3.67
Haptic experience	4	4	2	4	3	3.5	5	3	4	3	3	3	3.44

For the visual rendering of the NP and bones, volunteers gave scores ranging between 3 and 4. According to the feedback on those scores of 3, there were some differences between the system’s visual effects and real scenes, although virtual objects were distinguishable. Participant 3 gave the lowest score of 2 and reflected rendering is unsatisfactory so he can only roughly tell the objects and scenes.

For scores on understanding of the operation, TELD-SSPS received the best scores. Though 2 participants found limited guidance during actual surgical maneuvers, others felt the prototype was effective in helping them understand intraoperative procedures. Moreover, the two participating doctors pointed out that the system’s visual rendering could be further improved. They also recognized that the skills taught were basic and that further work is needed to fully simulate intricate surgical details.

For the haptic experience, most participants consider that the training system can reflect the force characteristics on simulated objects adequately. Only Participant 3 noted a mediocre haptic experience, mainly due to the lack of coordination between hand and eye. The tool seen was not aligned with the tool held, making it difficult to adapt to the operation.

## Discussion

To evaluate the method proposed, the NP model constructed is evaluated for three deformation-related characteristics, which are crucial for its clamping and removal operations. From the visual observation and data collected above, the model reasonably represents the biomechanical properties of the actual tissue during the plastic deformation, creeping, and stress relaxation, three typical effects of NP during the operations.

With the tissue and bones properly rendered, as a training system, TELD-SSPS is evaluated for its user experiences. Out of the three indices, the prototype got the best score (average: 3.67) in enhancing the understanding of the operation. And then followed by haptic experience and visual effect. From the users’ perspective, undergraduates and interns gave a higher score generally than doctors, while interns better recognized the system for its haptic (average: 4) and assistance in understanding the operation (average: 3.5) than undergraduates (average 3.5 and 3.2 accordingly). This indicates that the system is more beneficial for beginners’ learning and training, aligning with the original intent of this work. However, it is undeniable that for doctors skilled in TELD surgery, this method still has certain limitations and lacks completeness compared to actual surgery. There are differences between the method and real operations. TELD-SSPS needs further improvement in refining operations and optimizing model algorithms.

## Conclusions

This work focuses on simulating key TELD operations: clamping and removal of the NP. An optimized MPM is employed to simulate the tissue and then together with the bones are rendered into a virtual training environment, on top of these, a surgical training prototype system, TELD-SSPS, has been developed. The tissue modeling is evaluated by simulation experiments, which show the NP tissue model can effectively simulate the actual mechanical properties of NP and offer a realistic visual experience. The subjective assessments from the users of the prototype system are quite positive in terms of the visual and haptic feedback effect of the simulated operations. Moreover, the ability to enhance understanding of the operations got the best subjective evaluation, which proves the potential training benefits of the proposed approach. However, better training effects, visual rendering of the tissue, and integration of more surgical skills into the system need further development efforts.

## Data Availability

The datasets used and/or analyzed during the current study are available from the corresponding author on reasonable request.

## Abbreviations

MPM: Material point method
TELD: Transforaminal endoscopic lumbar discectomy
NP: Nucleus pulposus
VR: Virtual reality
PK: Piola-Kirchhoff
SSPS: Simulation prototype system
